# Suicidality of refugees and asylum seekers - prevalence rates in a global perspective and implications for global mental health

**DOI:** 10.1192/j.eurpsy.2026.10368

**Published:** 2026-07-31

**Authors:** E. Mittendorfer-Rutz

**Affiliations:** Department of Clinical Neuroscience, Karolinska Institutet, Stockholm, Sweden

## Abstract

**Background:**

Refugees and asylum seekers experience elevated exposure to pre‑migration trauma, dangerous migration journeys, and post‑migration stressors, all of which contribute to increased vulnerability to suicidal behaviour.

**Methods:**

The presentation will give a summary of recent meta analyses, systematic reviews and global health analyses and will present an outline on the implications on global mental health.

**Results:**

Rates of suicidal behaviour across displaced populations vary widely with significant heterogeneity across settings and methodologies. Asylum seekers consistently show higher suicide risk in comparative studies. Risk is also heightened among subgroups, including *unaccompanied minors*, and individuals facing prolonged detention, insecure residency, or disrupted access to care. In contrast, suicide rates in refugees with residence permits in high-income countries have in several studies reported to be lower than in the host population. Protective factors include *social support, family cohesion,* and individual *coping strategies.*

**Global Mental Health Implications:**

Forced displacement has created unprecedented global mental health burdens. Refugees and asylum seekers experience higher rates of depression, PTSD, psychosis, and suicidality due to adversity at each stage of migration. Barriers to mental health care—including language differences, stigma, restricted service access, and disrupted continuity of care—further increase suicide risk. Existing prevention strategies remain limited by insufficient evidence, with reviews highlighting gaps in data on comorbidities and intervention efficacy. Strengthening global mental health strategies will require coordinated, culturally grounded, evidence‑based approaches aimed at early identification, accessible care, and multisectoral suicide prevention.

**Disclosure of Interest:**

None Declared

